# The Impact of Transurethral Enucleation Therapeutic Approach in All-Size Benign Prostatic Obstruction Pathology: From Contemporary Technological Advances to Evidence-Based Clinical Progresses

**DOI:** 10.3390/diagnostics15040416

**Published:** 2025-02-08

**Authors:** Catalin-Andrei Bulai, Razvan-Dragos Multescu, Petrisor-Aurelian Geavlete, Ana Maria Andreea Punga, Adrian Militaru, Bogdan-Gabriel Buzescu, Cosmin-Victor Ene, Cristian Mares, Bogdan-Florin Geavlete

**Affiliations:** 1 Department of Urology, “Carol Davila” University of Medicine and Pharmacy, 050474 Bucharest, Romania; catalin.bulai@umfcd.ro (C.-A.B.); ana.punga13@gmail.com (A.M.A.P.); militaru.adrian21@yahoo.com (A.M.); bogdanmbuzescu@yahoo.com (B.-G.B.); cosmin85_ene@yahoo.com (C.-V.E.); dr.marescristian@gmail.com (C.M.); bogdan.geavlete@umfcd.ro (B.-F.G.); 2 Department of Urology, “Saint John” Clinical Emergency Hospital, 042122 Bucharest, Romania

**Keywords:** transurethral enucleation, benign prostatic obstruction, laser enucleation, minimally invasive urology, bipolar enucleation

## Abstract

Transurethral enucleation (TUE) has revolutionized the management of benign prostatic obstruction (BPO), offering a minimally invasive solution with superior efficacy across all prostate sizes. This review explores the advancements in TUE techniques, including Holmium Laser Enucleation of the Prostate (HoLEP), Thulium Laser Enucleation of the Prostate (ThuLEP), and bipolar enucleation, highlighting their clinical benefits, safety profiles, and long-term outcomes. Compared to traditional approaches such as transurethral resection of the prostate (TURP) and open prostatectomy, TUE has been associated with reduced complication rates, shorter recovery times, and durable symptom relief. The manuscript also examines patient-centered considerations, such as quality of life improvements and preservation of sexual function, which contribute to high patient satisfaction. Furthermore, the economic advantages of TUE, driven by reduced retreatment rates and healthcare costs, underscore its value for both patients and healthcare systems. Emerging innovations, including artificial intelligence (AI), promise to further enhance procedural planning, surgical precision, and training pathways. Efforts to expand global access through cost-effective adaptations like bipolar enucleation and targeted training initiatives are paving the way for wider adoption of TUE. With its adaptability, technological advancements, and focus on patient outcomes, TUE is poised to become the global standard of care in BPO management.

## 1. Introduction

Benign prostatic obstruction (BPO), a prevalent urological condition characterized by the enlargement of the prostate gland, significantly affects the quality of life for millions of men worldwide [1]. As the prostate enlarges, it can compress the urethra, leading to bothersome lower urinary tract symptoms (LUTS) such as frequent urination, weak urine flow, difficulty initiating or stopping urination, and nocturia [2]. The prevalence of BPO increases with age, making it a substantial public health concern [3]. Beyond its impact on individual patients, BPO imposes a considerable burden on healthcare systems, necessitating frequent medical consultations, diagnostic tests, and various treatment modalities [4].

Historically, the management of BPO has relied on a combination of surgical and non-surgical approaches. Medical therapies, such as alpha-blockers and 5-alpha reductase inhibitors, can alleviate symptoms by relaxing the prostate muscle and reducing prostate size, respectively [5]. However, these medications often provide only partial relief and may not be suitable for all patients [6]. Surgical interventions, such as Transurethral Resection of the Prostate (TURP), have long been considered the gold standard for treating BPO. TURP involves the removal of excess prostate tissue using a specialized instrument inserted through the urethra [7]. While TURP can effectively alleviate symptoms, it is associated with potential complications, including bleeding, urinary incontinence, and retrograde ejaculation [8]. Moreover, TURP may not be suitable for patients with large prostate glands or those who have undergone previous pelvic surgeries [9].

In recent years, transurethral enucleation (TUE) has emerged as a pivotal and highly efficacious modality in the surgical management of BPO, redefining standards of care and clinical outcomes [10]. This technique involves the precise removal of enlarged prostate tissue through the urethra, utilizing advanced technology to achieve superior surgical outcomes [11]. Unlike traditional TURP, which removes tissue incrementally, TUE allows for the complete removal of the prostate adenoma in one piece [12]. This approach not only facilitates thorough treatment but also provides critical pathological specimens for accurate diagnosis [7].

Transurethral enucleation offers significant advantages over traditional BPO surgeries. It is adaptable to all prostate sizes, including very large glands exceeding 80 cc, making it an effective alternative to open prostatectomy [13]. Secondly, TUE is associated with a lower risk of bleeding due to the meticulous control of blood vessels during enucleation [14]. This results in shorter catheterization times, reduced hospital stays, and fewer perioperative complications [15]. Finally, TUE delivers durable relief from LUTS, significantly enhancing patient quality of life and minimizing the need for retreatment [12].

The emergence of TUE techniques, including bipolar enucleation and laser enucleation methods such as Holmium Laser Enucleation of the Prostate (HoLEP) and Thulium Laser Enucleation of the Prostate (ThuLEP), represents a significant advancement in the management of BPO. These approaches have established new standards for safety, efficacy, and patient satisfaction [16]. They offer minimally invasive alternatives to open prostatectomy for large prostates [17] and have demonstrated superior long-term outcomes and more favorable complication profiles compared to TURP [18]. Moreover, the availability of diverse techniques enables clinicians to tailor treatments to individual patient needs, optimizing outcomes [19].

As our understanding of BPO and its management evolves, the adoption of TUE is expected to expand globally, providing a safe, effective, and minimally invasive solution for millions of men affected by this common condition. Advances in surgical instrumentation, imaging, and surgeon training will continue to refine these techniques, ensuring their central role in modern urological practice [20].

A comprehensive literature search was conducted to identify relevant studies on TUE techniques, focusing on technological advancements, clinical outcomes, and patient-centered considerations. The following electronic databases were used: PubMed, Scopus, Web of Science, and Embase. The search covered studies published from January 2010 to December 2023 to capture the latest developments in the field. The search strings included combinations of the following terms: “transurethral enucleation” OR “TUE”, “Holmium Laser Enucleation of the Prostate” OR “HoLEP”, “Thulium Laser Enucleation of the Prostate” OR “ThuLEP”, “Thulium Fiber Laser” OR “pulsed Thulium:YAG laser”, “benign prostatic hyperplasia” OR “BPH” OR “benign prostatic obstruction”, “minimally invasive urology”. Studies were included if they reported on clinical outcomes, technological innovations, training techniques, or economic analyses related to TUE. Articles not in English, conference abstracts, and studies lacking sufficient methodological details were excluded.

This narrative review aims to thoroughly examine the advancements, clinical benefits, and future directions of transurethral enucleation of the prostate (TUE), emphasizing its impact on patient outcomes, healthcare systems, and global accessibility. By summarizing the current evidence, we aim to provide a comprehensive perspective on this evolving field.

## 2. Technological Advances in Transurethral Enucleation

### 2.1. Development of Contemporary TUE Techniques

The development of transurethral enucleation techniques represents a significant advancement in the management of BPO. Among the most notable approaches are HoLEP, ThuLEP, and bipolar enucleation of the prostate (BEP). These methods leverage technological innovations to enhance precision, safety, and clinical outcomes.

Holmium Laser Enucleation of the Prostate employs a 2.140 nm laser, recognized for its cutting and coagulation capabilities. Studies suggest that HoLEP facilitates the complete removal of prostate adenomas, potentially reducing bleeding risks and retreatment rates. Its versatility has made it applicable across various prostate sizes, although the steep learning curve remains a notable challenge. Advances in structured training programs have contributed to broader adoption [21,22].

Thulium Laser Enucleation of the Prostate employs a continuous-wave laser with a shorter wavelength, enhancing vaporization and soft tissue precision. It is associated with excellent hemostasis, reduced operative times, and outcomes comparable to HoLEP. The method is gaining popularity due to its potential for smoother tissue removal [23]. Thulium Fiber Laser (TFL) and pulsed Thulium:YAG lasers represent the next generation of advancements in transurethral enucleation. TFL utilizes a novel fiber-based laser delivery system, offering greater precision, enhanced vaporization, and efficient tissue ablation with minimal collateral damage. Its ability to deliver higher energy densities at lower power settings reduces heat spread, making it particularly suitable for delicate tissues [24].

Pulsed Thulium:YAG lasers, on the other hand, combine the cutting efficiency of Holmium lasers with the continuous vaporization properties of Thulium lasers. These devices have shown promising results in improving operative times, hemostasis, and procedural safety. Early studies suggest that they may provide advantages in treating large prostates and challenging anatomical cases [25].

Recent advancements in transurethral enucleation techniques include approaches like the “early-apical release” and the “omega sign technique”. The early-apical release technique enhances procedural precision by focusing on apical dissection early in the procedure, which helps maintain critical anatomical landmarks. This approach was shown to reduce the risk of residual adenoma and improve surgical efficiency [26]. The omega sign technique leverages specific anatomical markers to guide enucleation, providing a structured framework for tissue removal. This method has been associated with improved safety and reduced learning curves for surgeons adopting TUE techniques [27,28].

Bipolar enucleation utilizes electrosurgical energy in saline to perform cutting and coagulation. Evidence indicates that this approach minimizes fluid absorption risks while achieving outcomes comparable to laser techniques. Its shorter learning curve and cost-effectiveness make it an appealing option for surgeons transitioning from traditional TURP [29].

### 2.2. Technological Innovations Driving Progress

Recent innovations have further refined these techniques. Improved lasers with optimized wavelengths and power settings, coupled with real-time imaging systems, are reported to enhance procedural safety and efficacy. Additionally, the development of advanced morcellation devices has streamlined adenoma removal, reducing operative times and complication risks.

Technological advancements have contributed to significant progress in TUE, with potential improvements in efficacy and safety. Among these innovations, laser technology stands at the forefront. Modern lasers, such as Holmium and Thulium lasers, feature optimized wavelengths and adjustable power settings, which are designed to enhance precision in tissue dissection while minimizing collateral damage. These improvements not only facilitate complete adenoma removal but also ensure superior coagulation, reducing intraoperative bleeding and the need for transfusions [25].

Real-time imaging techniques have further augmented the surgeon’s ability to visualize anatomical structures during procedures. High-definition endoscopic cameras and advanced optical systems provide unparalleled clarity, allowing for precise identification of tissue planes and vital structures. These technologies reduce the likelihood of complications while increasing procedural confidence, particularly in challenging cases involving large prostates or previous surgeries [19].

Enhanced surgical instruments and morcellation devices have significantly improved the efficiency and safety of transurethral enucleation procedures. Modern morcellators, such as the Piranha and DrillCut systems, demonstrate higher tissue removal rates, reducing operative times and minimizing complications like tissue spillage. Ergonomically designed enucleation tools with improved handling also contribute to shorter operative times and better outcomes [30].

**Figure 1:**
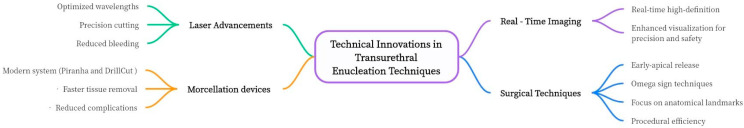
Technical innovations in transurethral enucleation techniques.

Technical innovations in transurethral enucleation techniques have significantly advanced procedural precision, safety, and efficiency. These advancements, including laser improvements, novel surgical approaches, real-time imaging systems, and modern morcellation devices, are summarized in Figure 1.

### 2.3. Training and Surgeon Expertise

The adoption and effective execution of transurethral enucleation techniques demand a significant level of surgical expertise due to their technical complexity. Procedures such as HoLEP and ThuLEP have a well-documented learning curve, requiring surgeons to gain proficiency in tissue dissection, anatomical landmark identification, and the operation of advanced surgical instruments [31]. For many, the initial phase of training may be challenging, with the potential for longer operative times or incomplete enucleation. However, with proper guidance and practice, these hurdles can be overcome, allowing surgeons to achieve the full benefits of TUE for their patients [32]. Simulation and hands-on training programs play a pivotal role in bridging the gap between theory and practice. High-fidelity simulation tools, including virtual reality platforms and anatomical models, provide a risk-free environment that may help surgeons refine their skills. These training modalities allow for repetitive practice, exposure to diverse surgical scenarios, and a focus on precision, all critical for mastering the nuanced techniques required for successful TUE. Complementing these simulations, hands-on workshops under expert mentorship provide real-world experience and immediate feedback. This structured approach is reported to enhance technical capabilities and build confidence, potentially preparing surgeons to handle complex cases in clinical settings [33]. The combination of simulation-based training and progressive involvement in live surgeries is reported to support a smooth transition from novice to independent practice. Moreover, ongoing education and skill enhancement through advanced courses, case reviews, and performance assessments help surgeons stay updated on the latest innovations and best practices for TUE. As the demand for minimally invasive solutions for BPO grows, structured and accessible training pathways are likely to be integral to expanding the global reach of TUE techniques, supporting high-quality care for patients worldwide [33].

## 3. Evidence-Based Clinical Outcomes

### 3.1. Clinical Benefits of TUE Approaches

Transurethral enucleation techniques have demonstrated exceptional clinical benefits across all prostate sizes, making them a highly versatile option for BPO. Unlike conventional methods, TUE ensures the complete removal of the adenoma, translating into durable symptom relief and improved patient outcomes. Studies have reported improvements in International Prostate Symptom Score (IPSS) and quality of life (QoL) measures following TUE. These benefits are sustained over the long-term, with reduced retreatment rates compared to other modalities [34]. Short-term outcomes further highlight the advantages of TUE. Postoperative catheterization times are markedly reduced, often to less than 24 h, reflecting faster recovery and reduced patient discomfort. Hospital stays are often shortened, with many patients discharged within one or two days of the procedure. Studies suggest that the meticulous control of blood vessels during enucleation may minimize intraoperative bleeding and reduce the need for blood transfusions. These favorable postoperative results suggest the potential of TUE as a minimally invasive alternative for BPO management [35]. While TUE offers remarkable benefits as a standalone approach, its true value becomes evident when compared to conventional surgical methods such as transurethral resection of the prostate and open prostatectomy.

### 3.2. Comparison to Other Surgical Methods

Transurethral enucleation (TUE) techniques, including Holmium Laser Enucleation of the Prostate (HoLEP) and Thulium Laser Enucleation of the Prostate (ThuLEP), have demonstrated significant advantages over traditional surgical methods such as transurethral resection of the prostate (TURP) and open prostatectomy. While TURP has long been considered the standard of care for benign prostatic obstruction (BPO), it is less effective in managing larger prostate volumes and is associated with a higher risk of complications, including bleeding and retrograde ejaculation. In contrast, TUE techniques allow for complete adenoma removal, providing superior symptom relief and reducing retreatment rates [36].

Compared to open prostatectomy, traditionally used for large prostates exceeding 80 cc, TUE offers a minimally invasive alternative with comparable efficacy. Patients undergoing TUE experience shorter operative times, faster recovery, and fewer perioperative complications, such as reduced blood loss and shorter catheterization times. These benefits position TUE as a safer and more patient-friendly option, especially in cases involving large prostate volumes [37].

Further comparisons have been made between laser technologies within TUE, including Holmium, Thulium, and Thulium Fiber Lasers. Systematic reviews and meta-analyses have shown that HoLEP remains the most extensively studied and widely adopted technique, offering durable symptom relief, reduced retreatment rates, and a favorable complication profile. However, ThuLEP demonstrates comparable efficacy, particularly for medium-sized prostates, with shorter operative times due to its continuous-wave laser mechanism that enhances vaporization and hemostasis [19,37].

Emerging evidence on Thulium Fiber Laser (TFL) highlights its potential to further optimize procedural outcomes. TFL’s unique fiber-based laser delivery system enables precise energy application with minimal thermal spread, reducing the risk of collateral tissue damage. Studies suggest that TFL delivers higher energy densities at lower power settings, making it especially suitable for complex cases, such as large prostates or patients on anticoagulation therapy. These findings underscore the adaptability and safety of newer laser technologies in expanding the clinical scope of TUE [11,38].

In addition, network meta-analyses comparing enucleation techniques to TURP and other laser-based approaches, such as photoselective vaporization, indicate that TUE provides more complete tissue removal and superior pathological specimens for diagnosis. These findings reaffirm the versatility of TUE techniques in addressing diverse clinical scenarios and prostate sizes while reducing perioperative risks and improving long-term outcomes [19,37].

These advantages, combined with its adaptability across prostate sizes, highlight TUE as a versatile option in modern urological practice (Table 1).

### 3.3. Impact on Large-Sized Prostates

Managing large prostate volumes (>80 cc) presents unique challenges, often requiring extensive tissue removal to alleviate symptoms effectively. Historically, open prostatectomy was the treatment of choice for such cases, but it is associated with significant morbidity and extended recovery times [42]. TUE, particularly HoLEP, has emerged as a promising alternative in this context [43].

TUE techniques have consistently demonstrated their efficacy in treating large prostates. The ability to enucleate the entire adenoma in a single procedure ensures complete symptom relief and significantly reduces the need for secondary interventions. Recent evidence from a propensity-score matching study has provided valuable insights into the comparative outcomes of HoLEP and Robot-Assisted Simple Prostatectomy (RASP) for managing large prostates. The study demonstrated that both techniques are effective for achieving significant improvements in symptom relief and quality of life metrics. However, HoLEP was associated with shorter operative times, reduced blood loss, and quicker postoperative recovery compared to RASP. These findings underscore HoLEP’s potential advantages as a minimally invasive alternative for treating large prostate volumes, while also highlighting the utility of RASP in cases where HoLEP may not be feasible due to anatomical or technical considerations [44]. Studies have shown that patients with large prostates achieve comparable or superior outcomes to those with smaller glands, with improvements in IPSS, QoL, and urinary flow rates. Additionally, the minimally invasive nature of TUE minimizes the risks traditionally associated with managing large prostate volumes, such as bleeding and prolonged hospital stays [13].

### 3.4. Complication Profiles

Transurethral enucleation techniques, such as HoLEP, have been associated with a favorable complication profile compared to traditional surgical methods like TURP and open prostatectomy. Systematic reviews and meta-analyses indicate that TUE may reduce the incidence of complications, including urinary incontinence and urethral strictures, while enhancing patient recovery outcomes [11]. The meticulous surgical approach of TUE is associated with a lower incidence of postoperative complications, including urinary incontinence and urethral strictures. Research indicates that TUE is linked to reduced incidence of complications, thereby improving patient safety and recovery outcomes [43]. Furthermore, TUE techniques have been associated with reduced perioperative morbidity, including lower rates of bleeding and shorter hospital stays, compared to traditional methods. This contributes to a more favorable recovery profile for patients undergoing TUE procedures [37]. Strategies to further reduce complications have focused on surgeon training and the use of advanced technologies. Proper training in anatomical dissection and enucleation techniques helps avoid damage to surrounding tissues, preserving urinary and sexual function. Technological advancements, such as real-time imaging and improved morcellation devices, have also played a critical role in ensuring safer outcomes. Overall, the combination of precise surgical methods and ongoing innovation has positioned TUE as one of the safest and most effective options for managing BPO [45].

## 4. Patient-Centered Considerations

### 4.1. Impact on Quality of Life

Transurethral enucleation techniques have consistently demonstrated significant improvements in QoL for patients suffering from BPO. Beyond the resolution of bothersome LUTS, TUE approaches are associated with high patient satisfaction rates due to their ability to preserve key functional outcomes. Sexual function, often a major concern for patients undergoing BPO interventions, is generally maintained with TUE techniques. Unlike traditional methods such as TURP, which are associated with higher rates of retrograde ejaculation, TUE is reported to reduce this risk, particularly when performed with precision [34].

Urinary retention, another distressing complication for many patients, is effectively addressed by TUE. Rapid postoperative recovery and shorter catheterization times enable patients to regain normal bladder function quickly, reducing their dependence on catheters and enhancing overall satisfaction. Furthermore, the durability of symptom relief provided by TUE translates into lasting improvements in daily activities and a return to a better quality of life. Patient-reported outcome measures, including improved IPSS and quality of life indices, consistently highlight the benefits of TUE over alternative treatments, affirming its role as a patient-centric therapeutic option [35].

### 4.2. Cost-Effectiveness

Holmium Laser Enucleation of the Prostate is associated with economic advantages for both patients and healthcare systems. While the initial costs of HoLEP procedures may be higher than those of conventional surgeries such as Transurethral Resection of the Prostate, the long-term cost savings are substantial. Studies suggest that HoLEP may be more cost-effective than TURP across all severities of benign prostatic hyperplasia. Additionally, HoLEP is associated with lower combined 5-year follow-up costs compared to TURP, due to its high durability and low retreatment rates [46]. These savings stem from reduced rates of retreatment and lower incidences of postoperative complications. For instance, studies have shown that the durable symptom relief achieved by TUE minimizes the need for additional interventions, sparing patients from recurrent medical expenses and disruptions to their lives [47]. From a healthcare system perspective, TUE contributes to cost-effectiveness by reducing hospitalization durations and resource utilization. Patients undergoing TUE typically experience shorter operative times, decreased hospital stays, and fewer postoperative complications, which translate into lower direct and indirect costs [16]. Additionally, the ability of TUE to handle large prostate volumes without necessitating open prostatectomy provides further economic benefits by avoiding more invasive and resource-intensive procedures. In resource-limited settings, innovations in training and access to cost-effective bipolar enucleation techniques have expanded the availability of TUE, making it a financially viable solution for a broader patient population [48].

### 4.3. Indications and Patient Selection

The success of TUE relies heavily on appropriate patient selection and tailored treatment planning. Candidates for TUE are typically those experiencing moderate to severe LUTS caused by BPO, particularly when medical therapy has proven ineffective or contraindicated. Patients with large prostates (>80 cc) may benefit from TUE, as its ability to achieve complete enucleation is associated with improved outcomes compared to other surgical options [13]. Individualizing treatment is essential for maximizing the benefits of TUE. Factors such as prostate size, comorbidities, patient preferences, and functional goals should guide decision-making. For younger patients or those particularly concerned about preserving sexual function, certain TUE techniques were developed to minimize the risk of retrograde ejaculation. For instance, the en-bloc and bladder neck preservation technique during HoLEP has demonstrated efficacy in maintaining antegrade ejaculation. Additionally, modified surgical approaches and non-ablative techniques have emerged with the aim of preserving antegrade ejaculation. A systematic review highlighted various methods designed to maintain ejaculatory function while effectively treating benign prostatic obstruction [49]. Similarly, patients with a history of previous pelvic surgeries or comorbid conditions such as anticoagulation therapy can benefit from the precision and reduced bleeding risks associated with TUE [39].

Shared decision-making between patients and clinicians plays a central role in ensuring that treatment aligns with the patient’s expectations and lifestyle. Educating patients about the benefits, risks, and long-term outcomes of TUE empowers them to make informed choices. As the availability of TUE continues to grow globally, its versatility and adaptability make it an increasingly valuable option for diverse patient populations [50].

**Figure 2:**
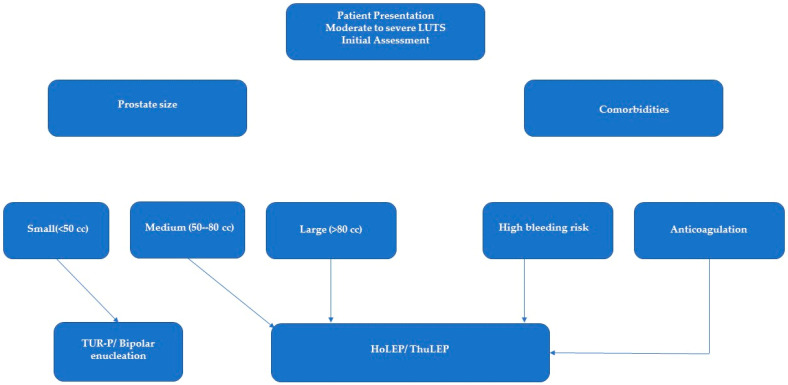
Treatment selection algorithm for patients with moderate-to-severe LUTS caused by BPO.

The successful management of BPO relies on appropriate patient selection and individualized treatment planning. Factors such as prostate size, comorbidities, bleeding risks, and patient preferences play a central role in determining the most suitable intervention. For instance, TURP and bipolar enucleation are typically reserved for small to medium prostates (<50 cc), whereas HoLEP and ThuLEP are preferred for larger glands (>80 cc). Patients with high bleeding risk or anticoagulation therapy may benefit from laser-based techniques like HoLEP and ThuLEP due to their superior hemostatic properties. The treatment decision-making process is summarized in the algorithm below (Figure 2)**.**

## 5. Future Directions

### 5.1. Ongoing Research and Innovations

The field of transurethral enucleation is undergoing constant evolution, with ongoing research driving the development of innovative techniques and technologies to enhance patient outcomes. One promising area of exploration is the refinement of laser systems used in TUE, such as advancements in Holmium and Thulium lasers. These innovations are intended to enhance precision, potentially reduce operative times, and minimize complications such as bleeding [24]. Emerging laser technologies with variable power settings and improved tissue interaction hold the potential to make procedures more efficient and accessible, particularly for complex cases involving large prostates [51]. Investigational therapies are also being explored to complement existing TUE techniques. Novel energy-based tissue ablation methods, such as waterjet-based or ultrasound-guided approaches, are under evaluation for their ability to further reduce invasiveness while maintaining efficacy [52]. Additionally, new morcellation devices with enhanced safety profiles are being developed to streamline tissue removal and reduce the risk of complications [53]. These technologies not only aim to improve the procedural experience for both patients and surgeons but also to expand the applicability of TUE to a broader range of clinical scenarios.

Research efforts increasingly focus on long-term outcomes and quality of life measures, aiming to refine patient selection criteria and optimize treatment protocols. Comparative trials investigating the efficacy of TUE against emerging alternatives continue to solidify its position as a leading solution for BPO [40].

### 5.2. Expanding Accessibility

Despite its numerous advantages, access to TUE remains limited in resource-constrained settings due to the high costs of equipment and the need for specialized training. Efforts to broaden the availability of TUE are underway, with initiatives focused on reducing barriers to adoption. One approach has been the development of cost-effective alternatives, such as bipolar enucleation techniques, which utilize widely available electrosurgical units instead of more expensive laser systems. These adaptations make TUE more accessible to healthcare facilities with limited resources, ensuring that a greater number of patients can benefit from its superior outcomes [54]. Policy-level interventions are critical to driving the global uptake of TUE. Governments and healthcare organizations must prioritize investments in training programs and subsidize advanced surgical equipment to enable widespread adoption [55]. International collaborations and partnerships between high-resource and low-resource regions can facilitate knowledge transfer, equipment sharing, and the establishment of training centers [56]. Efforts by professional urological societies to standardize training and certification for TUE techniques are also vital in ensuring the safe and effective dissemination of this technology. Expanding access to TUE has the potential to improve individual patient outcomes and alleviate the economic burden on healthcare systems by reducing the need for retreatment and long hospital stays. As these accessibility initiatives gain traction, TUE is poised to become a standard of care worldwide [57].

### 5.3. Integration of Artificial Intelligence (AI)

The integration of artificial intelligence (AI) into surgical practice represents an exciting frontier for TUE. AI is expected to enhance training, improve procedural planning, and increase the precision of surgical execution. AI-powered training simulators, incorporating machine learning algorithms, can provide personalized feedback to trainees, accelerating the learning curve and ensuring consistent competency among surgeons. These platforms can simulate complex cases and rare scenarios, enabling surgeons to practice and refine their skills in a risk-free environment [58]. During the planning phase, AI-driven imaging analysis can assist in preoperative assessment by identifying anatomical variations and predicting potential complications. Advanced imaging tools, powered by AI, can also help map the prostate gland and surrounding structures with greater accuracy, enabling surgeons to tailor the procedure to the specific needs of the patient. This level of customization not only improves outcomes but also reduces the risk of adverse events [59].

Artificial intelligence is revolutionizing surgical procedures by enhancing precision and reducing errors through real-time guidance and data analytics. AI-assisted navigation systems provide surgeons with real-time feedback, improving accuracy and safety during operations. The integration of AI with robotic systems further refines surgical movements, ensuring optimal enucleation while preserving vital structures. This combination aims to enable more precise and controlled surgical interventions, potentially minimizing the risk of damage to surrounding tissues [60]. Moreover, AI-enabled data analytics continuously monitor procedural metrics, offering actionable insights to improve future performance and outcomes. By analyzing data from previous surgeries, AI systems can identify patterns and suggest improvements, leading to enhanced surgical techniques and patient care [61].

As AI technology continues to advance, its role in TUE will likely expand, driving innovation and setting new standards for safety, efficacy, and efficiency. By combining the precision of AI with the expertise of surgeons, TUE has the potential to achieve unprecedented levels of success in BPO management.

Transurethral enucleation techniques have consistently demonstrated significant improvements.

## 6. Conclusions

Transurethral enucleation represents a significant advancement in the management of BPO, combining clinical efficacy with patient-centered benefits and potential economic advantages. Its ability to completely remove prostatic adenomas with minimal invasiveness provides durable symptom relief and improved quality of life for patients across a range of prostate sizes, including larger glands. Compared to traditional methods, TUE has been associated with reduced complications, retreatment rates, and hospitalization times, positioning it as a promising therapeutic option.

TUE’s adaptability continues to evolve with advancements in technology, including laser systems and bipolar techniques, which aim to enhance precision and safety. The integration of artificial intelligence into TUE practices holds the potential to revolutionize preoperative planning, intraoperative guidance, and surgeon training, further refining procedural outcomes and expanding accessibility to diverse patient populations.

Efforts to standardize training and broaden access to TUE, particularly in resource-constrained settings, remain critical to its global adoption. Policy-level initiatives to subsidize equipment and provide targeted training programs are essential for ensuring equitable availability. As accessibility initiatives and technological advancements progress, TUE is poised to become an increasingly valuable option in BPO management, offering safe, effective, and potentially economically viable solutions for patients worldwide.

## Figures and Tables

**Table 1:** Comparison table for TUE techniques.

Parameter	HoLEP	ThuLEP	BEP
Efficacy (IPSS, QoL, Q_max_)	High, durable symptoms relief [35,39]	High, comparable to HoLEP [25,36]	Moderate, effective for smaller prostate [29,40]
Complication rate	Low (bleeding, incontinence minimal) [35,39]	Low [25,36]	Moderate [29,40]
Prostate size sustainability	Large (>80 cc) [36,39]	Medium–large (50–80 cc) [25,36]	Small–Medium (<50 cc) [29,40]
Learning curve	Steep [39,41]	Moderate [25,36]	Short [29]
Technology	Laser-based [35,39]	Laser-based, efficient energy delivery [25]	Uses standard electrosurgical units [40]
Cost	High [39,41]	Moderate [25]	Low [40]

IPSS—International Prostate Symptom Score; QoL—Quality of Life; Qmax—maximum urinary flow rate; cc—cubic centimeters.

## Data Availability

No new data were generated or analyzed in this study. Data sharing is not applicable to this article as it is based on a review of the existing literature.
